# Effectiveness of Multicomponent Interventions in Office-Based Workers to Mitigate Occupational Sedentary Behavior: Systematic Review and Meta-Analysis

**DOI:** 10.2196/44745

**Published:** 2023-07-26

**Authors:** Liying Zhou, Xinxin Deng, Kangle Guo, Liangying Hou, Xu Hui, Yanan Wu, Meng Xu, Yongsheng Wang, Shanshan Liang, Kehu Yang, Xiuxia Li

**Affiliations:** 1 Health Technology Assessment Center/Evidence-Based Social Science Research Center School of Public Health Lanzhou University Lanzhou China; 2 Evidence-Based Medicine Center School of Basic Medical Sciences Lanzhou University Lanzhou China; 3 Key Laboratory of Evidence-Based Medicine and Knowledge Translation of Gansu Province Lanzhou China; 4 Infection Management Section Gansu Provincial Hospital Lanzhou China

**Keywords:** sedentary behavior, workplace, multicomponent, systematic review

## Abstract

**Background:**

Sedentary time in workplaces has been linked to increased risks of chronic occupational diseases, obesity, and overall mortality. Currently, there is a burgeoning research interest in the implementation of multicomponent interventions aimed at decreasing sedentary time among office workers, which encompass a comprehensive amalgamation of individual, organizational, and environmental strategies.

**Objective:**

This meta-analysis aims at evaluating the effectiveness of multicomponent interventions to mitigate occupational sedentary behavior at work compared with no intervention.

**Methods:**

PubMed, Web of Science, EMBASE, and Cochrane Central Register of Controlled Trials (CENTRAL) databases were searched from database inception until March 2023 to obtain randomized controlled trials (RCTs) assessing the efficacy of multicomponent interventions on occupational sedentary behavior among office-based workers. Two reviewers independently extracted the data and assessed the risk of bias by using the Cochrane Collaboration’s risk of bias tool. The average intervention effect on sedentary time was calculated using Stata 15.1. Mean differences (MDs) with 95% CIs were used to calculate the continuous variables. Subgroup analyses were performed to determine whether sit-stand workstation, feedback, and prompt elements played an important role in multicomponent interventions. Further, the GRADE (Grading of Recommendations, Assessment, Development, and Evaluation) system was used to evaluate the certainty of evidence.

**Results:**

A total of 11 RCTs involving 1894 patients were included in the analysis. Five studies were rated as low risk of bias, 2 as unclear risk of bias, and 4 as high risk. The meta-analysis results showed that compared with no intervention, multicomponent interventions significantly reduced occupational sitting time (MD=–52.25 min/8-h workday, 95% CI –73.06 to –31.44; *P*<.001) and occupational prolonged sitting time (MD=–32.63 min/8-h workday, 95% CI –51.93 to –13.33; *P*=.001) and increased occupational standing time (MD=44.30 min/8-h workday, 95% CI 23.11-65.48; *P*<.001), whereas no significant differences were found in occupational stepping time (*P*=.06). The results of subgroup analysis showed that compared with multicomponent interventions without installment of sit-stand workstations, multicomponent interventions with sit-stand workstation installment showed better effects for reducing occupational sitting time (MD=–71.95 min/8-h workday, 95% CI –92.94 to –51.15), increasing occupational standing time (MD=66.56 min/8-h workday, 95% CI 43.45-89.67), and reducing occupational prolonged sitting time (MD=–47.05 min/8-h workday, 95% CI –73.66 to –20.43). The GRADE evidence summary showed that all 4 outcomes were rated as moderate certainty.

**Conclusions:**

Multicomponent interventions, particularly those incorporating sit-stand workstations for all participants, are effective at reducing workplace sedentary time. However, given their cost, further research is needed to understand the effectiveness of low-cost/no-cost multicomponent interventions.

## Introduction

For most working adults, the majority of their sedentary time is accrued during work time [1,2]. More sitting time was reported at work than in any other sitting activity such as watching television or using a computer at home on weekdays. Studies also revealed that the sitting time of work for full-time office workers accounted for approximately 60%-90% of the total daily sitting time in a workday [3,4]. In addition, there is evidence that working adults who spend long periods of time sitting at work do not necessarily compensate for their sitting by being more active outside work [5]. It is crucial to note that contemporary research indicates that excessive sedentary behavior is detrimentally linked to many health-related risks such as cardiovascular disease, unhealthy aging, musculoskeletal disorders, poor bone health, poor metabolic health, and all-cause mortality, especially when sedentary time accumulates in prolonged uninterrupted bouts [6,7]. The workplace has been highlighted by the World Health Organization as a vital setting for health promotion actions to reduce sedentary behavior [8].

According to 2 umbrella reviews, the utilization of electronic and mobile health tools such as mobile apps is associated with a reduction in sedentary behavior [9,10]. In addition, the recent umbrella reviews indicate that interventions targeting the physical environment, specifically the implementation of active workstations, represent the most efficacious category of interventions for mitigating sedentary behavior in workplaces [11,12]. Following the first randomized controlled trial (RCT) in 2013 [13], a surging multitude of subsequent RCTs has been undertaken to evaluate the efficacy of multicomponent interventions in mitigating sedentary behavior in workplaces among office-based employees [14,15]. The multicomponent interventions encompass a comprehensive amalgamation of diverse modalities, including individual strategies such as counselling, prompts, telephonic support, and motivational interviewing; environmental strategies such as active workstations, prompting posters, exercise sessions, and access to a gym; as well as organizational strategies such as workshops, site visits, consultations, and appointing team leaders, ambassadors, or management support. Currently, there is a burgeoning research interest for implementing multicomponent interventions aimed at mitigating sedentary behavior among office workers [16].

With the growing public health concerns surrounding sedentary behavior in the workplace among nonmanual employees, it is important to determine the effectiveness of multicomponent interventions for office-based workers. This is not only from a theoretical and best practice perspective but also due to the lack of a systematic summary on this topic. Multicomponent interventions are designed to address multiple levels of influence for sustainable behavior change, which aligns with the socioecological theory that emphasizes targeting multiple levels of influence [17]. Despite this, to date, evidence on the efficacy of multicomponent interventions has not been systematically summarized. Previous literature reviews on this topic have been constrained in their scope, as they have not concentrated specifically on the efficacy of multicomponent interventions in mitigating sedentary behavior. In addition, these reviews are outdated and have incorporated only a small number of RCTs in their analyses, which restricts the capacity to arrive at conclusive determinations regarding their impact [18,19].

Thus, this systematic review and meta-analysis includes newly published RCTs and aims to identify the effect of multicomponent interventions on sedentary workplace behavior based on more sedentary-related outcomes such as occupational sitting time, occupational standing time, occupational stepping time, and prolonged occupational sitting time. Furthermore, given that electronic and mobile health tools and physical environment interventions such as the implementation of an active workstation are independent and effective components affecting sedentary time, we performed subgroup analyses based on whether these components were included in the multicomponent interventions and attempted to identify the more effective components of multicomponent interventions.

## Methods

### Search Strategy

PubMed, Web of Science, EMBASE, and Cochrane Central Register of Controlled Trials (CENTRAL) databases were searched from database inception to March 1, 2023. The main search strategies were as follows: (occupation* or workplace* or employe* or office* or work-site or worker* or staff* or white-collar*) AND (sedentary or sitting or inactivity or “physical activity” or “physically active”) AND (random* or blind* or singleblind* or doubleblind* or tripleblind* or RCT* or control*). The detailed search strategy is presented in Multimedia Appendix 1. In addition, the World Health Organization International Clinical Trials Registry Platform search portal, ClinicalTrials.gov, conference materials, and reference lists (backward and forward) of the studies identified using the above search strategy were searched manually for additional studies on March 1, 2023. We also searched relevant grey literature, including clinical guidelines, reports, and working papers through Google and grey literature databases [20].

### Inclusion and Exclusion Criteria

Studies were included if they met all the eligibility criteria mentioned in Table 1.

**Table 1:** Inclusion and exclusion criteria for study selection.

	Inclusion criteria	Exclusion criteria
Participants	Workers aged ≥18 years who work in the office, do jobs that generally do not involve manual labor, or wear uniforms or work clothes were included	People working in the transportation industry (such as taxi drivers, truck drivers, bus drivers, and airline pilots) and those who operate heavy equipment (such as crane operators and bulldozer operators) were excluded
Intervention	Multicomponent interventions integrating individual strategies (eg, counselling, prompts, telephonic support, motivational interviews), environmental strategies (eg, active workstations, prompting posters, exercise sessions, access to a gym), and organizational strategies (eg, workshops, site visits, consultations, and appointment of team leaders, ambassadors, management support) aimed at changing sedentary behavior were eligible	Single-component interventions (such as implementation of a sit-stand workstation alone) or 2-level interventions consisting of only 2 types of strategies of the multicomponent intervention were excluded
Comparison	Usual work practices or waitlist	Other interventions such as single-component interventions, 2-level interventions, or multicomponent interventions were excluded
Outcome	Workplace sitting and activity outcomes during work hours: occupational sitting time, occupational standing time, occupational stepping time, and occupational prolonged sitting time as measured by self-report (eg, questionnaires) or using objective measures (eg, accelerometer) at the primary end point	Studies that reported daily sitting or activity outcomes during total waking hours rather than workplace sitting and activity outcomes specifically during work hours were excluded
Study design	Only studies with a parallel control (or treatment-comparison group) such as randomized controlled trials, controlled trials, cluster randomized controlled trials, or quasi-experimental studies were included in this review	Reviews, expert opinions, meta-analyses, or before-and-after studies were excluded
Language	English	Non-English

### Study Selection and Data Extraction

Two independent reviewers performed title screening and data extraction of the retrieved studies, and any conflicts were resolved through a discussion. We used EndNote X9.1 software to omit duplicates. Subsequently, based on the inclusion and exclusion criteria, the 2 reviewers screened the titles and abstracts to discard irrelevant studies. Studies were removed from further review if both reviewers excluded them. Otherwise, full papers were obtained for a detailed review. We extracted the following data from the included studies by using a prespecified data form: general information (publication date, name of the first author, and study country/region), study population (age, gender, education, and employment status), multicomponent intervention (component, frequency, and duration of intervention; delivery mode; theoretical framework; status of sit-stand workstation installation; and inclusion of feedbacks and prompts), comparison intervention (waitlist or no intervention), outcomes (occupational sitting time, occupational standing time, occupational stepping time, and occupational prolonged sitting time [sitting time accumulated in bouts ≥30 minutes]), and follow-up time.

### Risk of Bias Assessment

The Cochrane risk of bias tool was used to evaluate the quality of RCTs based on randomization and allocation concealment (selection bias), blinding of personnel and participants (performance bias), blinding of outcome assessment (detection bias), incomplete outcome data (attrition bias), selection of reported results (reporting bias), and sources (other bias), and the RCTs were evaluated as having low, high, or unclear risk of bias [21]. Studies were rated as having a low risk of bias if all items were low risk. When 1 item was high risk, the study was rated as having a high risk of bias. The studies that did not fall into the abovementioned categories were rated as having an unclear risk of bias [22-24]. In all the included studies, blinding of the participants or personnel to the intervention and allocation concealment was not possible in accordance with the nature and aim of the interventions, wherein intervention participants typically underwent changes in environment (such as installation of sit-stand workstation). Therefore, the performance bias item and allocation concealment item were excluded from the bias assessment. However, for the allocation concealment item, trials were assessed as high risk if there was a contamination between intervention and control group participants, that is, if participants from the same office or company ended up in different groups. Participants in the control group are more likely to be less sedentary under the influence of individuals in the intervention group in the same office, regardless of group allocation [25-27]. Studies were considered to have a low risk of bias if measures were taken to minimize contamination, such as using cluster trials or assigning intervention and control participants to separate floors in the same building. Studies were classified as unclear risk of bias if there was insufficient information to determine the presence of either of the above conditions. For studies with multiple publications, we reviewed all relevant papers, including protocol papers, to ensure that the quality of the trial was judged on all available information.

### Data Treatment and Statistical Analysis

Data synthesis was based on the general recommendations from the Cochrane Handbook for Systematic Reviews of Interventions [28]. Both the adjusted mean difference (AMD) and its SD were extracted for studies that reported AMD for the intervention and control groups after the intervention or change (change from baseline or change score) for time spent in sedentary behavior. For studies that did not report AMD or change, the unadjusted MD and SD were calculated from the means and SDs at baseline and after follow-up in each group. Therefore, the AMDs and unadjusted MDs were pooled together using the inverse variance method. For studies with multiple intervention arms, estimates were combined into a single group as a weighted average using Cochrane formula for combining subgroups if all the intervention arms are multicomponent. If, in a multiple arm trial, an arm was not a multicomponent intervention, then we excluded that arm.

We used minutes per 8 hours of work time at the workplace as the standard unit in the main group and subgroup analyses because it was the most commonly reported unit of measurement in the included trials. We converted all the measurement units for sitting at work into min/8-h workday, where needed and possible and assumed the data referred to a 5-day work week if this was not reported. Therefore, studies that reported min/8-h workday were combined with studies that reported min/d, min/workday, h/d, and min/week to measure the overall minutes in sedentary behavior per workday.

MD value with 95% CI based on the inverse variance method was used as the summary statistic for continuous variables, including sitting time, standing time, stepping time, and prolonged sitting time. Heterogeneity was evaluated using the Higgins *I*^2^ value, and values ≤50% and >50% were considered to indicate low and high heterogeneity, respectively. MD values and the corresponding 95% CIs were calculated. *P*<.05 was considered to indicate statistical significance [29]. All statistical analyses were performed using Stata version 15.1 (StataCorp LLC). The random effects model was used. As mentioned in the Introduction, electronic and mobile health tools and physical environment interventions, especially installation of active workstations, are independent and effective components affecting sedentary time. Therefore, we performed subgroup analyses to determine whether sit-stand workstations, feedbacks (individual feedback on sedentary behavior at baseline and following the monitoring of activity during the trial), and prompts (individual prompts to regularly break up sitting through email, telephone, computer software, or prompting app) played an important role in multicomponent interventions. With regard to the sit-stand workstation element, we divided the included studies into 2 subgroups based on whether individual sit-stand workstations were installed in the trial: multicomponent intervention with sit-stand workstation installment and multicomponent intervention without sit-stand workstation installation. Based on prior research highlighting the significant influence of availability on the utilization of sit-stand workstation [30], we made the decision to mandate the complete availability of the sit-stand workstation within the subgroup of multicomponent interventions that involved the installation of such workstations. To maintain categorization consistency, the studies including workplaces that provide some sit-stand workstations available for shared use were classified into the subgroup of multicomponent intervention without sit-stand workstation installment. In the case of shared active workstations, office workers were required to temporarily vacate their original workstations to utilize shared facilities. In these instances, shared sit-stand workstation interventions were paired with other environmental strategy interventions such as access to a gym and the provision of company bikes but not with individual sit-stand workstations. Sensitivity analyses were performed on the outcome indicators of ≥10 studies to explore their potential sources and assess the robustness of these results. Egger test was used to assess publication bias [31].

### Certainty Assessment

Two reviewers independently rated the certainty of evidence associated with specific outcomes and constructed a table summarizing the certainty of evidence findings using the GRADE (Grading of Recommendations, Assessment, Development, and Evaluation) system [32,33]. The GRADE approach uses 5 domains, namely, risk of bias, inconsistency, indirectness, imprecision, and publication bias, which are assessed to determine the degree of confidence in the estimate of effect or association derived from the meta-analysis. Prespecified level of certainty was initially considered based on the study design (eg, high certainty for RCTs). Correspondingly, following the rating by reviewers, the certainty of evidence was changed and divided into no downgrade (not serious), downgrade by 1 level (serious), or downgrade by 2 levels (very serious). Finally, the certainty of evidence of each outcome was judged as different levels of evidence, that is, high, moderate, low, and very low.

## Results

### Study Selection

**Figure 1:**
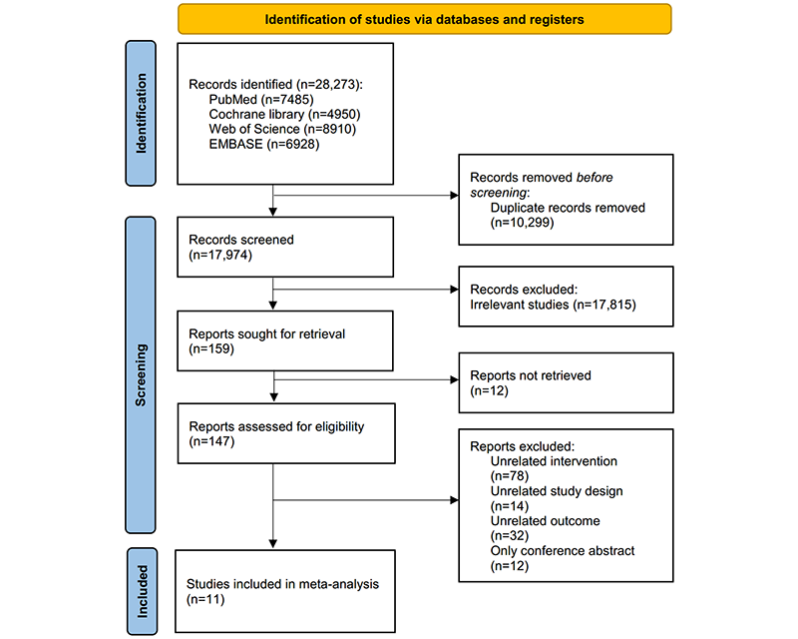
Flowchart of the selection of randomized controlled trials that assessed the effects of multicomponent interventions on the occupational sedentary behavior of office-based workers.

A flow diagram of the literature selection process is shown in Figure 1. A total of 28,273 relevant records were initially identified, of which 10,299 were excluded because of duplication. The titles and abstracts were screened, and 17,815 were deemed unsuitable. After reading the full text, another 159 studies were excluded because of inappropriate study design or topic of research. Finally, 11 studies were included in this meta-analysis [13-15,34-41].

### Main Characteristics of the RCTs

Of the 11 studies [13-15,34-41] included in this meta-analysis, 7 were cluster RCTs [15,34,35,37,39-41] and 4 were quasi-RCTs [13,14,36,38] (Multimedia Appendix 2). Regarding the location of the studies, 4 were conducted in Australia [13,14,36,37], 3 in England [34,35,39], and 1 each in Denmark [15], Taiwan [38], Sweden [40], and the Netherlands [41]. The studies were published between 2013 and 2022. All the trials compared the multicomponent intervention with no intervention among office-based workers. Multicomponent interventions aiming at reducing sedentary time in the workplace consisted of individual strategies, environmental strategies, and organizational strategies. In Multimedia Appendix 3 [13-15,34-41], a detailed account of the constituent elements and implementation particulars of multicomponent interventions implemented in each of the trials is presented. Seven of the 11 studies [13-15,34-37] had environmental components that implemented interventions to install sit-stand workstations, whereas the other 4 [38-41] did not. The studies included 1894 participants (1127 and 767 in the intervention and control groups, respectively) with a mean age ranging between 37.3 and 49.5 years. The sample size largely varied among the studies and ranged between 25 and 756. The intervention and follow-up periods varied from 1 month to 12 months. Seven studies [13, 14, 34, 35, 37, 39, 41] assessed sedentary behavior outcomes using the activPAL accelerometer, 3 studies [15, 36, 40] used the ActiGraph accelerometer, and only 1 [38] study used self-reported methods (ie, a self-efficacy scale). The dropout rates in the studies ranged from 1.98% to 35.52%.

### Risk of Bias Assessment

**Figure 2:**
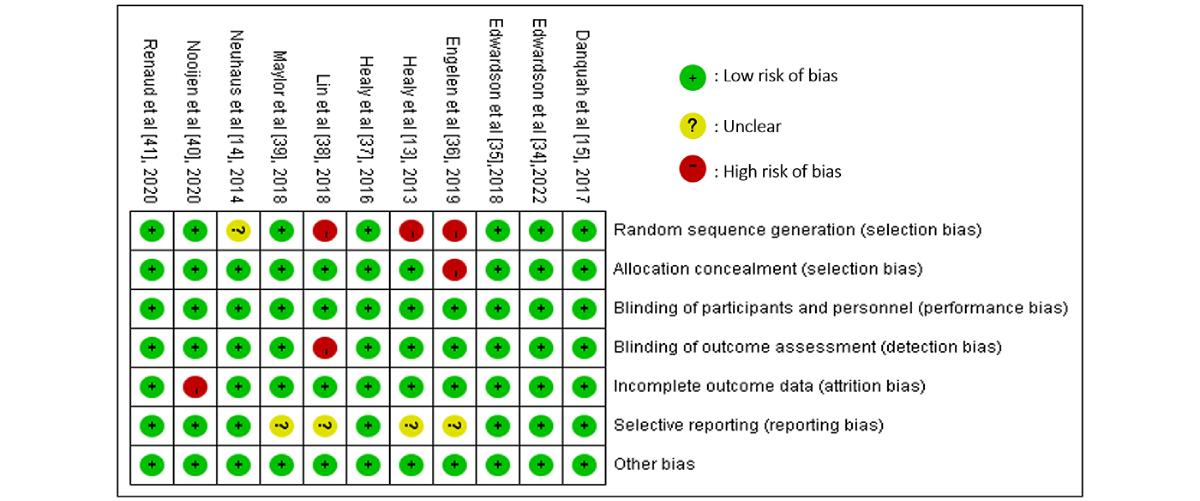
Risk of bias summary: the judgments of the reviewers about each risk of bias item for each of the included 11 randomized controlled trials using the Cochrane risk of bias tool [13-15,34-41].

As shown in Figure 2, the risk of bias was high in 4 studies [13,36,38,40], unclear in 2 [14,39], and low in 5 [15,34,35,37,41]. Regarding the random sequence generation assessment, 3 studies [13,36,38] did not adhere to random sequence generation, and thus we judged them to have a high risk of bias. Additionally, 1 trial [14] was assessed as unclear risk of bias because it gave no information about how randomization was done. For allocation concealment, 1 trial [36] was assessed as high risk due to contamination between the intervention and control group participants. Regarding outcome assessment, 1 trial [38] was rated as high risk of bias because its outcome measures were self-reported. Regarding incomplete outcome data, 1 study [40] was assessed as high risk of bias due to attrition rates of more than 25%. Regarding selection of the reported results, 4 trials [13,36,38,39] were assessed as unclear risk of bias due to there being no available trial registration or published protocol.

### Summary of the Intervention Effects

#### Occupational Sitting Time

**Figure 3:**
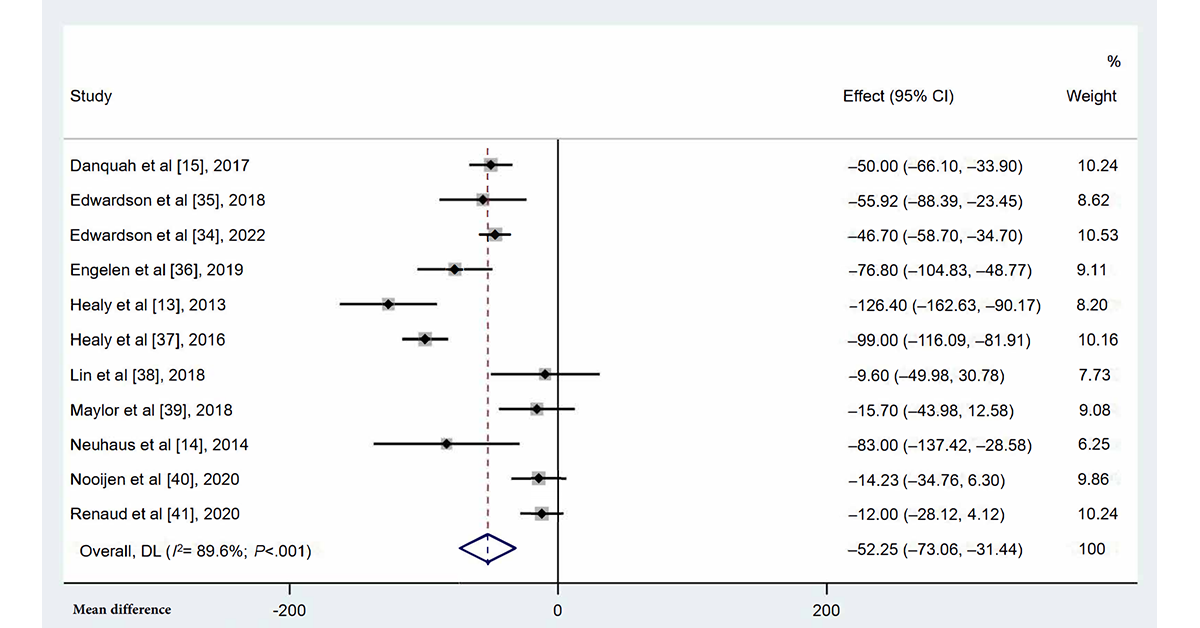
Forest plots for the effects of multicomponent interventions on occupational sitting time in office-based workers compared with usual practices [13-15,34-41]. Note: Weights are from random-effects model. DL: DerSimonian-Laird approach.

All 11 included studies, with the multicomponent intervention and control groups including 1127 and 767 individuals, respectively, measured occupational sitting time (Figure 3). Participants in the multicomponent intervention groups had significantly shorter occupational sitting time than those in the control groups (MD=–52.25 min/8-h workday, 95% CI –73.06 to –31.44; *I*^2^=89.6%; *P*<.001). Sensitivity analysis showed that no single study significantly affected the overall heterogeneity.

#### Occupational Standing Time

**Figure 4:**
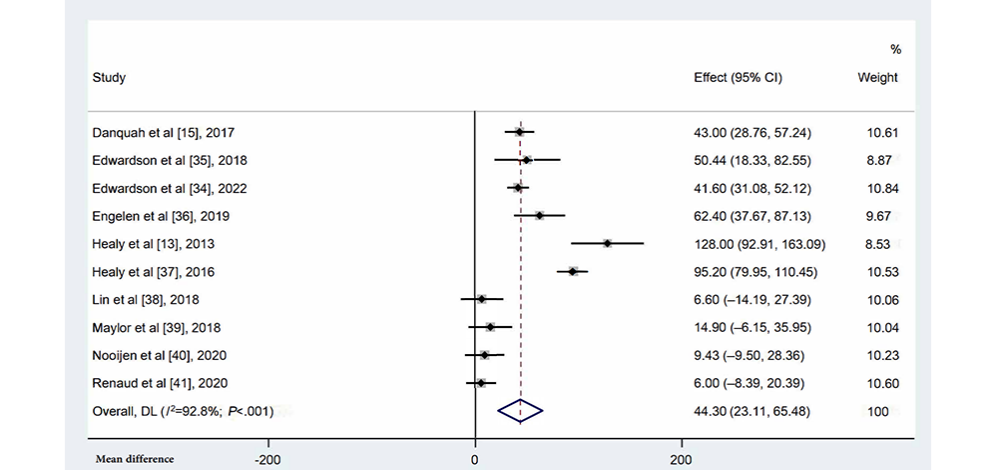
Forest plots for the effects of multicomponent interventions on occupational standing time in office-based workers compared with usual practices [13,15,34-41]. Note: Weights are from random-effects model. DL: DerSimonian-Laird approach.

A total of 10 studies [13,15,34-41] with the multicomponent intervention and control groups including 1115 and 754 individuals, respectively, measured occupational standing time at the workplace (Figure 4). Participants who were in the multicomponent intervention group had significantly increased standing time compared with those in the control group (MD=44.30 min/8-h workday, 95% CI 23.11-65.48; *I*^2^=92.8%; *P*<.001). Sensitivity analysis showed that no single study significantly affected the overall heterogeneity.

#### Occupational Stepping Time

**Figure 5:**
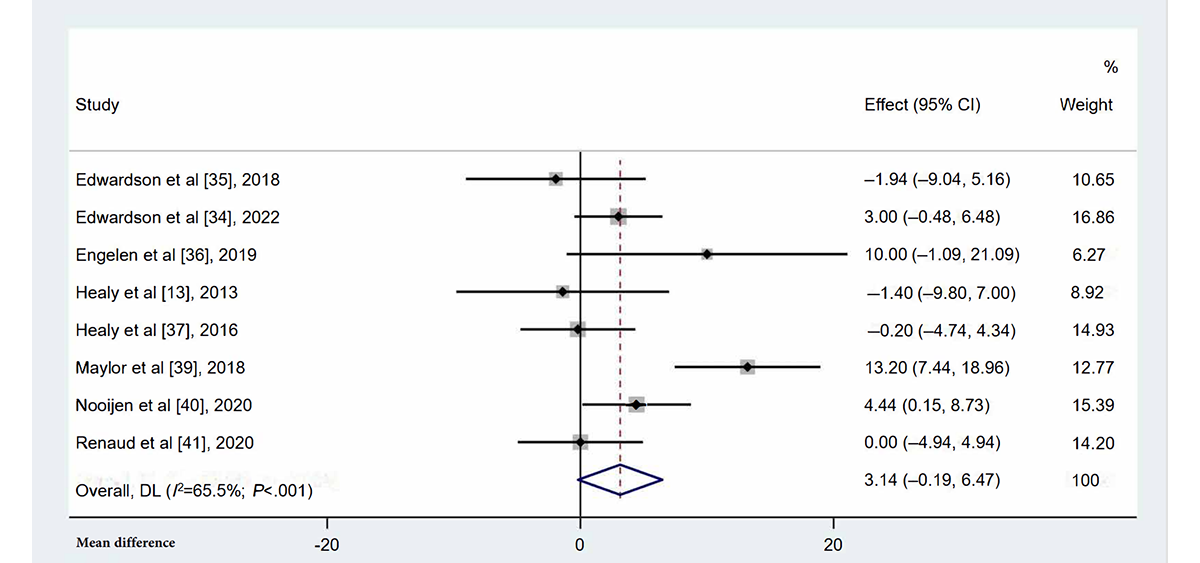
Forest plots for the effects of multicomponent interventions on occupational stepping time in office-based workers compared with usual practices [13,34-37,39-41]. Note: Weights are from random-effects model. DL: DerSimonian-Laird approach.

Of the 11 studies [13-15,34-41], 8 [13,34-37,39-41] reported the occupational stepping time of individuals (Figure 5). No statistically significant difference in occupational stepping time was observed between the 2 groups (MD=3.14 min/8-h workday, 95% CI –0.19 to 6.47; *I*^2^=65.5%; *P*=.06).

#### Occupational Prolonged Sitting Time

**Figure 6:**
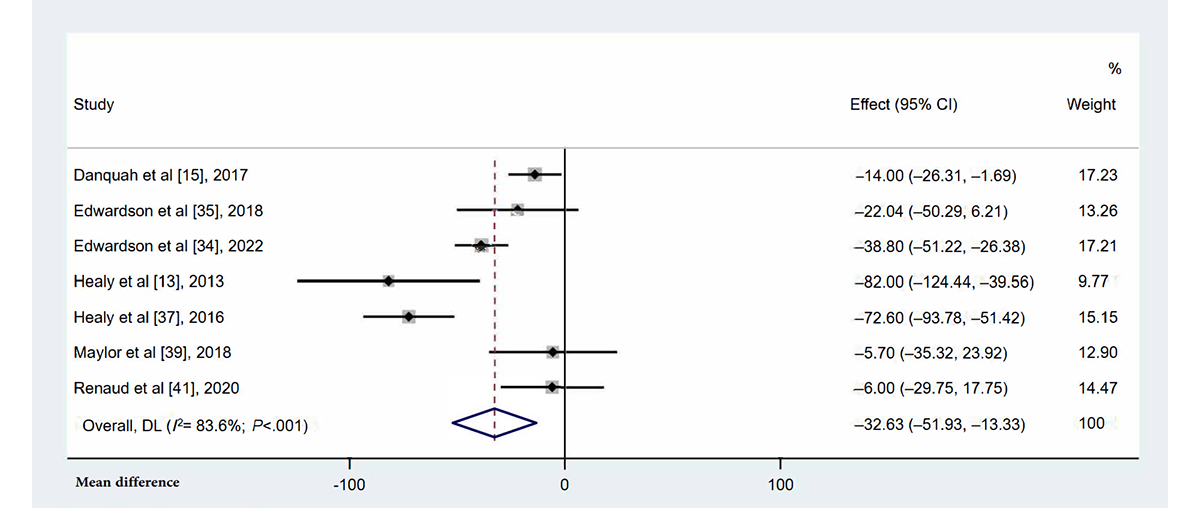
Forest plots for the effects of multicomponent interventions on occupational prolonged sitting time in office-based workers compared with usual practices [13,15,34,35,37,39,41]. Note: Weights are from random-effects model. DL: DerSimonian-Laird approach.

Seven studies [13,15,34,35,37,39,41] reported a reduction in occupational prolonged sitting time at work following multicomponent interventions (Figure 6). Multicomponent interventions significantly reduced prolonged sitting time (MD=–32.63 min/8-h workday, 95% CI –51.93 to –13.33; *I*^2^=83.6%; *P*=.001).

#### Subgroup Analyses

In the subgroup analyses for installment of sit-stand workstations during multicomponent interventions, the multicomponent intervention subgroup with sit-stand workstation installment showed considerable reduction in the occupational sitting time (MD=–71.95 min/8-h workday, 95% CI –92.94 to –51.15; *I*^2^=84.2%; *P*<.001); however, the multicomponent intervention subgroup without sit-stand workstation installment showed a smaller reduction in the occupational sitting time (MD=–14.25 min/8-h workday, 95% CI –22.78 to –5.72; *I*^2^=0%; *P*=.001). Furthermore, the results showed no heterogeneity in the subgroup without the installment of sit-stand workstations. The multicomponent intervention subgroup with the sit-stand workstation installment showed a great increase in the occupational standing time (MD=66.56 min/8-h workday, 95% CI 43.45-89.67; *I*^2^=89.9%; *P*<.001), but the multicomponent intervention subgroup without the sit-stand workstation installment showed no such effect (MD=6.82 min/8-h workday, 95% CI –0.09 to 13.67; *I*^2^=0%; *P*=.06). The results showed no heterogeneity in the subgroup analysis without the installment of sit-stand workstations. Further, the multicomponent intervention subgroup with the installment of sit-stand workstations showed a considerable reduction in occupational prolonged sitting time (MD=–47.05 min/8-h workday, 95% CI –73.66 to –20.43; *I*^2^=88.7%; *P*=.001), but the multicomponent intervention subgroup without the installment of a sit-stand workstation showed a smaller reduction (MD=–11.49 min/8-h workday, 95% CI –22.78 to –0.19; *I*^2^=0%; *P*=.04). On the contrary, the multicomponent intervention subgroup with the installment of sit-stand workstations showed no significant difference in stepping time (MD=1.53 min/8-h workday, 95% CI –0.90 to 3.96; *I*^2^=5.8%; *P*=.22), but the subgroup without the installment of sit-stand workstations showed increased occupational stepping time (MD=5.22 min/8-h workday, 95% CI 0.31-10.13; *I*^2^=74.9%; *P*=.04). On performing subgroup analysis based on inclusion of prompt and feedback elements in multicomponent interventions, no significant differences between subgroups were observed overall (Multimedia Appendix 4).

#### Publication Bias

**Figure 7:**
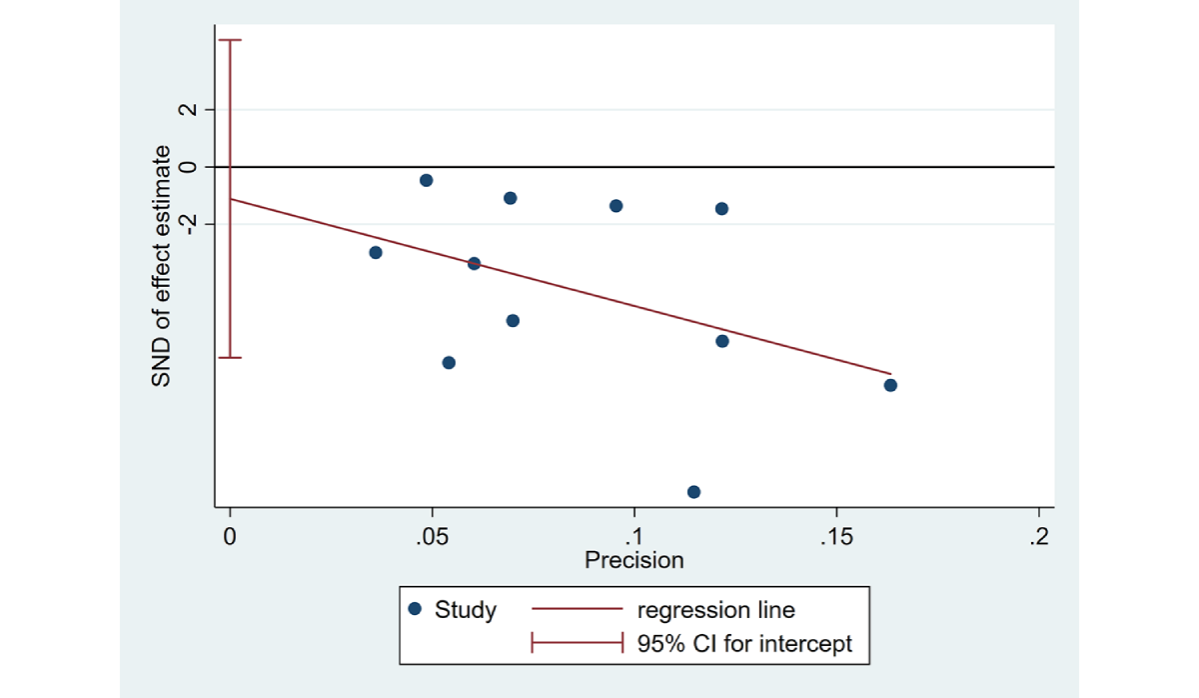
Egger publication bias plot for assessing publication bias among the 11 included randomized controlled trials. SND: standard normal deviation.

A funnel plot based on Egger test was constructed, and no publication bias was found (*P*=.68) (Figure 7).

#### Certainty Assessment

The GRADE evidence summary of certainty showed that all 4 outcomes were rated as moderate certainty (occupational sitting time, occupational standing time, occupational stepping time, and occupational prolonged sitting time). The main reason we downgraded the certainty of evidence was limitations in study design, that is, high risk of bias in the studies, including nonrandomized allocation sequence, possible contamination between the intervention and control groups, self-reported outcome measures, a high rate of loss to follow-up, and unclear risk of bias in the studies, including no information about random allocation sequence generation, trial registration, or published protocol (Multimedia Appendix 5).

## Discussion

### Principal Findings

In this meta-analysis of RCTs, we included 11 studies [13-15,34-41] involving 1894 participants from 6 countries. The analyses showed, with moderate certainty of evidence, that multicomponent interventions were effective in reducing occupational sitting time, increasing occupational standing time, and reducing occupational prolonged sitting time. We did not find a statistically significant difference in the occupational stepping time outcome between the groups. Based on subgroup analysis results, multicomponent interventions with the installment of sit-stand workstations were significantly more effective in decreasing both occupational sitting time (*P*<.001) and occupational prolonged sitting time (*P*<.001) while increasing occupational standing time (*P*<.001). Conversely, the subgroup without the sit-stand workstation installment demonstrated only marginal reduction in occupational sitting time and occupational prolonged sitting time but demonstrated a minor increase in occupational stepping time. Minimal differences were observed between multicomponent intervention subgroups with the presence and absence of feedbacks and prompts in occupational sitting time, standing time, stepping time, and prolonged sitting time. More RCTs are still needed to strengthen the evidence body.

### Potential Interpretations of the Findings

These findings may be explained by multicomponent interventions implemented in the included studies, which reduced sedentary behavior primarily by increasing sitting-to-standing transitions by using a sit-stand workstation rather than by increasing stepping [42]. Multicomponent interventions may primarily reduce occupational sitting and occupational prolonged sitting time and increase occupational standing time by promoting the use of sit-stand workstations among office workers in their environmental strategies through individual and organizational strategies. The provision of a sit-stand workstation permits continued work at a computer while standing as opposed to encouraging regular ambulation [43]. Therefore, sit-stand workstation installation may be required to significantly reduce occupational sitting time. However, the subgroup without the installment of sit-stand workstations exhibited only slight reductions in both occupational sitting time (MD=–14.25 min/8-h workday) and occupational prolonged sitting time (MD=–11.49 min/8-h workday) and a minor increase in occupational stepping time (MD=5.22 min/8-h workday). In addition, it should be noted that the increase in occupational stepping time associated with an 8-hour workday is minimal, and the effect size is negligible. Furthermore, the increased occupational sitting time only constitutes a small fraction of the overall reduction in occupational sitting time. The impact of increased occupational stepping time on reducing sedentary behavior in the workplace is limited. Consequently, we deduce that some multicomponent interventions implementing measures such as step/pedometer challenges, walking routes and resources, walking meetings, and lunch walks to reduce sedentary behaviors demonstrated limited effectiveness in increasing the stepping time of participants because most of these measures were of secondary and facilitative nature throughout the multicomponent intervention [35,44]. Moreover, the effectiveness of these measures may also be limited by space, feasibility, and convenience. Specifically, all interventions reduced the occupational total sitting time and prolonged sitting time, and most increased occupational standing time by using sit-stand workstations. These changes occurred across the workday among participants, although there was a wide individual variability in these changes [45,46].

This explanation can also be confirmed by Danquah et al [15], who suggested that the multicomponent intervention was effective in reducing sitting time and prolonged sitting time and in increasing the number of sit-to-stand transitions among office workers who already have sit-stand workstations (which is a standard equipment at most Danish workplaces, introduced for ergonomic considerations) [15]. In addition, Renaud et al [41] concluded that the multicomponent intervention had little to no effect in reducing occupational sitting time, which may be because of the relatively low intensity of the intervention, that is, it only involved the replacement of 25% of sitting workstations with sit-stand workstations. In addition, Michaud et al [47] conducted a cluster-RCT comparing multicomponent interventions with and without the incorporation of a sit-stand workstation and reported that the former intervention, with the installment of a sit-stand workstation, resulted in a reduction in occupational sitting time as well as total sitting time, thus strongly supporting our findings. Therefore, we believe that sit-stand workstations play a central role in multicomponent interventions and that the other components play a role in facilitating and increasing the effect.

### Comparisons With Other Reviews

Our findings on the effects of multicomponent interventions are consistent with several other systematic reviews in this area, which have demonstrated the benefits of such interventions in reducing sedentary time among office workers [18,19]. However, in comparison with previous systematic reviews and meta-analyses that investigated the effectiveness of multicomponent interventions, we included 3 more outcomes (occupational standing time, occupational prolonged sitting time, and occupational stepping time). In addition, subgroup analyses, including the installation of sit-stand workstations, feedbacks, and prompts, were conducted to investigate the significant constituents of multicomponent interventions, predicated on the inclusion of sit-stand workstations, feedback, and prompt elements, which were found to be independent and efficacious factors influencing sedentary time in previous studies. In brief, we included 11 RCTs with 1894 participants, which was almost 3 times the number of RCTs and participants included in the previous meta-analyses. Nonetheless, we observed similar results suggesting that multicomponent interventions are effective in reducing sitting time. With a more robust analysis, we could conclude that multicomponent interventions are also effective in reducing prolonged sitting time and increasing standing time. Furthermore, we used the GRADE method to assess the certainty of the evidence for the effectiveness of multicomponent interventions in reducing occupational sitting time and occupational prolonged sitting time and increasing occupational standing time and occupational stepping time among office workers.

### Assessment of Evidence Quality

Although the results of this meta-analysis are based on rigorously designed RCTs, they should be interpreted with caution because of the risk of bias. The 5 study designs [15,34,35,37,41] identified to have a low risk of bias met all standards. The remaining 4 studies [13,36,38,40] were deemed to have a high risk of bias, and 2 [14,39] were considered to have unclear risk of bias. This was mainly demonstrated in the fact that the allocation sequence was not randomized; there was possible contamination between the intervention and control groups; there was a high rate of loss to follow-up; and there was no available information about random allocation sequence generation, trial registration, or published protocol. This may have affected the authenticity of the reported results. Allocation concealment and blinding of participants and personnel was not possible once the study was underway because of the open plan nature of the environmental strategies in multicomponent interventions. However, 1 trial was assessed as high risk because of contamination between the intervention and control group participants; in this trial, participants from the same office or company were placed in different groups [36]. Participants in the control group were likely to be less sedentary because of the influence of the intervention group participants in the same office [25-27]. Therefore, while allocation concealment and blinding are not possible, the certainty of evidence was also downgraded 1 level to moderate due to the other limitations in the study design, such as the allocation sequence not being randomized, possible contamination between the intervention and control groups, a high rate of loss to follow-up, and no information about random allocation sequence generation, trial registration, or published protocol. These methodological limitations underscore the importance of future clinical trials adhering to robust study design principles and implementation guidelines. RCTs with rigorous randomization procedures should be prioritized to minimize bias and increase the validity of findings [48]. Additionally, adopting the CONSORT (Consolidated Standards of Reporting Trials) as the reporting standard can significantly improve study quality and transparency [49]. CONSORT guidelines provide a structured framework for reporting essential aspects of trial design, conduct, and analysis, thereby enabling readers to evaluate the study’s validity and replicate the findings. To further enhance the scientific quality and reliability of RCTs, investigators should consider utilizing the Cochrane quality assessment tool [21]. This tool allows researchers to conduct a comprehensive self-examination of the study design, hypothesis formulation, data collection and analysis methods, and risk of bias assessment. By critically evaluating these aspects, researchers can identify and address potential limitations, thus strengthening the overall methodological rigor of the trial. Furthermore, the preregistration of studies and publishing research protocols can be beneficial for improving the transparency and trustworthiness of the evidence generated [50,51]. This practice helps reduce the risk of selective outcome reporting and ensures that the reported findings align with the initial study plan.

### Strengths and Limitations

This review was based on RCTs with real-world data. We conducted a comprehensive systematic search of 4 primary databases. Any differences between reviewers during screening, data extraction, risk of bias assessment, and evidence certainty grading were resolved through a discussion. We used 4 outcome measures to assess the effects of multicomponent interventions on sedentary behavior in the workplace. Estimates from the fully adjusted models in each study were used in our analyses to reduce the potential for confounding. We used the GRADE approach to assess the certainty of evidence, which will allow the readers to clearly understand the uncertainty in results. Furthermore, we conducted sensitivity analyses to test the robustness of the results. No evidence of publication bias was found. However, when interpreting the results, the following limitations should be considered. First, the countries included in this review were mainly high-income countries, thereby limiting the generalizability of the results. Office workers in high-income countries pay more attention to physical health than those in low-income countries; accordingly, participants may have shown higher motivation during the trial. Second, differences among office workers may affect the reliability of the evidence obtained in this study, such as job type, length of work, levels of physical and cognitive loads across sectors and industries, and workload. Third, the limited sample size observed in certain studies, coupled with variations in measurement units, likely exerted an influential impact on the wide CIs, leading to imprecise estimations of effect sizes. Furthermore, some included studies have been evaluated to possess high and unclear risk of bias due to limitations in study designs. In the future, more high-quality cluster-RCTs with large sample sizes are needed to strengthen the evidence body. Fourth, a substantial degree of heterogeneity was evident in the findings. However, we found no heterogeneity in the multicomponent intervention without the installment of a sit-stand workstation, whereas the multicomponent intervention with the sit-stand workstation installment showed heterogeneity. A high degree of heterogeneity was still observed in the subgroup of multicomponent intervention with the sit-stand workstation installment. Therefore, we speculate that the installment of a sit-stand workstation as well as its specific type and function (eg, whether it was electronically regulated) constituted the primary sources of heterogeneity. Regrettably, due to inadequate description of these parameters in the included studies, we were unable to conduct more comprehensive subgroup analyses or meta-regression analyses to further explore the origins of heterogeneity.

### Conclusions

Multicomponent interventions are indeed effective in reducing sedentary behavior in workplaces. In particular, multicomponent interventions including the component of sit-stand workstation installation are more effective in reducing sedentary behavior in workplaces. The reduction in sitting time was mainly replaced by standing time, which we presume primarily resulted from the increased use of the sit-stand workstation. Thus, the sit-stand workstation may be a key component of multicomponent interventions. Therefore, future research will benefit from assessing the relative contribution of different individual and organizational components for mitigating sedentary behavior in workplaces through factorial designs or multiple arms. Moreover, future studies should add detailed cost-effectiveness and time-of-day effect analyses while reporting multicomponent interventions. Further research to understand the effectiveness of low/no-cost multicomponent interventions is needed.

## Appendix

Multimedia Appendix 1 Detailed search strategy for PubMed, Web of Science, EMBASE, and Cochrane Central Register of Controlled Trials databases.

Multimedia Appendix 2 Basic characteristics of the included studies.

Multimedia Appendix 3 Detailed account of the constituent elements and implementation particulars of the multicomponent interventions implemented in each of the included trials.

Multimedia Appendix 4 Subgroup analyses of multicomponent intervention effects on the mitigation of occupational sedentary behavior based on the installation of sit-stand workstations, feedbacks, and prompts.

Multimedia Appendix 5 Summary of findings for multicomponent interventions for mitigating occupational sedentary behavior among office-based workers.

Multimedia Appendix 6 PRISMA checklist.

## Abbreviations

AMD: adjusted mean difference
CENTRAL: Cochrane Central Register of Controlled Trials
CONSORT: Consolidated Standards of Reporting Trials
GRADE: Grading of Recommendations, Assessment, Development, and Evaluation
MD: mean difference
RCT: randomized controlled trial

## Data Availability

The data set and any other materials of our study are available from the corresponding author on request.
