# Supplementary material for: Effectiveness of Multicomponent Interventions in Office-Based Workers to Mitigate Occupational Sedentary Behavior: Systematic Review and Meta-Analysis
Source: JMIR Public Health Surveill. 2023 Jul 26;9:e44745. doi: 10.2196/44745 (PMC10413238; doi:10.2196/44745)
Supplement: Multimedia Appendix 1 [file publichealth_v9i1e44745_app1.docx]

**Multimedia Appendix 1.** Detailed search strategy for PubMed, Web of Science, EMBASE, and Cochrane Central Register of Controlled Trials databases

Database: PubMed <1 March 2023>

Search Strategy:

| **#** | **Searches** | **Results** |
| --- | --- | --- |
| 1 | Occupation [Mesh] | 36,466 |
| 2 | Workplace [Mesh] | 29,060 |
| 3 | “Occupational Groups” [Mesh] | 708,744 |
| 4 | Occupation* [Title/Abstract] | 193,557 |
| 5 | Workplace* [Title/Abstract] | 53,804 |
| 6 | Employe* [Title/Abstract] | 491,095 |
| 7 | Office* [Title/Abstract] | 103,594 |
| 8 | (Work-site* [Title/Abstract]) OR (Worksite* [Title/Abstract]) | 5,055 |
| 9 | Worker* [Title/Abstract] | 223,589 |
| 10 | Staff* [Title/Abstract] | 204,088 |
| 11 | White-collar* [Title/Abstract] | 2,163 |
| 12 | OR/1-11 | 1,700,294 |
| 13 | “Sedentary Behavior” [Mesh] | 13,338 |
| 14 | “Sitting Position” [Mesh] | 1,438 |
| 15 | “Screen Time” [Mesh] | 1,089 |
| 16 | Sedentary [Title/Abstract] | 39,147 |
| 17 | Sitting [Title/Abstract] | 26,771 |
| 18 | (Inactivity [Title/Abstract]) OR (Inactive [Title/Abstract]) | 120,812 |
| 19 | (“Physical Activity” [Title/Abstract]) OR (“Physically Active” [Title/Abstract]) | 148,614 |
| 20 | (“Screen Time” [Title/Abstract]) OR (“Screen Behavior” [Title/Abstract]) OR (“Screen Behaviour” [Title/Abstract]) OR (“Screen Use” [Title/Abstract]) OR (“Screening Time” [Title/Abstract]) OR (“Screening Behavior” [Title/Abstract]) OR (“Screening Behaviour” [Title/Abstract]) OR (“Screening Use” [Title/Abstract]) | 6,131 |
| 21 | (“Computer Time” [Title/Abstract]) OR (“Internet Time” [Title/Abstract]) OR (“Computer Use” [Title/Abstract]) OR (“Internet Use” [Title/Abstract]) OR (“Computer Usage” [Title/Abstract]) OR (“Internet Usage” [Title/Abstract]) | 6,693 |
| 22 | OR/13-21 | 312,533 |
| 23 | "Randomized Controlled Trial" [Publication Type] | 588,897 |
| 24 | "Randomized Controlled Trials as Topic"[Mesh] | 164,422 |
| 25 | Random* [Title/Abstract] | 1,398,059 |
| 26 | (Blind* [Title/Abstract]) OR (Singleblind* [Title/Abstract]) OR (Doubleblind* [Title/Abstract]) OR (Trebleblind* [Title/Abstract]) OR (Tripleblind* [Title/Abstract]) | 343,814 |
| 27 | (RCT* [Title/Abstract]) OR (Control* [Title/Abstract]) | 4,563,920 |
| 28 | (“Clinical Trial” [Title/Abstract]) OR (“Clinical Trials” [Title/Abstract]) OR (“Clinical Study” [Title/Abstract]) OR (“Clinical Studies” [Title/Abstract]) OR (“Intention to Treat Analysis” [Title/Abstract]) | 637,130 |
| 29 | OR/23-28 | 5,877,378 |
| 30 | 12 AND 22 AND 29 | 7,485 |

Database: Cochrane Library <1 March 2023>

Search Strategy:

| **#** | **Searches** | **Results** |
| --- | --- | --- |
| 1 | MeSH descriptor: [Occupations] explode all trees | 243 |
| 2 | MeSH descriptor: [Workplace] explode all trees | 1,148 |
| 3 | MeSH descriptor: [Occupational Groups] explode all trees | 15,422 |
| 4 | (Occupation*):ti,ab,kw | 14,275 |
| 5 | (Workplace*):ti,ab,kw | 3,668 |
| 6 | (Employe*):ti,ab,kw | 20,464 |
| 7 | (Office*):ti,ab,kw | 12,384 |
| 8 | (Work-site*):ti,ab,kw OR (Worksite*):ti,ab,kw | 1,082 |
| 9 | (Worker*):ti,ab,kw | 13,417 |
| 10 | (Staff*):ti,ab,kw | 24,581 |
| 11 | (White-collar*):ti,ab,kw | 112 |
| 12 | OR/1-11 | 86,524 |
| 13 | MeSH descriptor: [Sedentary Behavior] explode all trees | 1,567 |
| 14 | MeSH descriptor: [Sitting Position] explode all trees | 220 |
| 15 | MeSH descriptor: [Screen Time] explode all trees | 62 |
| 16 | (Sedentary):ti,ab,kw | 9,326 |
| 17 | (Sitting):ti,ab,kw | 9,710 |
| 18 | (Inactivity):ti,ab,kw OR (Inactive):ti,ab,kw | 8,026 |
| 19 | (“Physical Activity”):ti,ab,kw OR (“Physically Active”):ti,ab,kw | 41,146 |
| 20 | (“Screen Time”):ti,ab,kw OR (“Screen Behavior”):ti,ab,kw OR (“Screen Behaviour”):ti,ab,kw OR (“Screen Use”):ti,ab,kw OR (“Screening Time”):ti,ab,kw | 717 |
| 21 | (“Screening Behavior”):ti,ab,kw OR (“Screening Behaviour”):ti,ab,kw OR (“Screening Use”):ti,ab,kw | 182 |
| 22 | (“Computer Time”):ti,ab,kw OR (“Computer Use”):ti,ab,kw OR (“Computer Usage”):ti,ab,kw | 237 |
| 23 | (“Internet Time”):ti,ab,kw OR (“Internet Use”):ti,ab,kw OR (“Internet Usage”):ti,ab,kw | 246 |
| 24 | OR/13-23 | 59,524 |
| 25 | MeSH descriptor: [Randomized Controlled Trial] explode all trees | 113 |
| 26 | (Random*):ti,ab,kw | 1,213,434 |
| 27 | (Blind*):ti,ab,kw OR (Singleblind*):ti,ab,kw OR (Doubleblind*):ti,ab,kw OR (Trebleblind*):ti,ab,kw OR (Tripleblind*):ti,ab,kw | 434,494 |
| 28 | (RCT*):ti,ab,kw OR (Control*):ti,ab,kw | 1,195,513 |
| 29 | (“Clinical Trial”):ti,ab,kw OR (“Clinical Trials”):ti,ab,kw OR (“Clinical Study”):ti,ab,kw OR (“Clinical Studies”):ti,ab,kw OR (“Intention to Treat Analysis”):ti,ab,kw | 654,842 |
| 30 | OR/#25-29 | 1,334,499 |
| 31 | #12 AND #24 AND #30 | 4,950 |

Database: Web of Science <1 March 20232>

All Web of Science Core Collection

Search Strategy:

| # | Searches | Results |
| --- | --- | --- |
| 1 | TS=(Occupation*) | 243,811 |
| 2 | TS=(Workplace*) | 98,209 |
| 3 | TS=(Employe*) | 1,183,892 |
| 4 | TS=(Office*) | 179,923 |
| 5 | (TS=(Work-site*)) OR TS=(Worksite*) | 5,689 |
| 6 | TS=(Worker*) | 336,151 |
| 7 | TS=(Staff*) | 217,740 |
| 8 | TS=(White-collar*) | 4,147 |
| 9 | OR/1-8 | 2,022,912 |
| 10 | TS=(Sedentary) | 48,999 |
| 11 | TS=(Sitting) | 83,351 |
| 12 | (TS=(Inactivity)) OR TS=(Inactive) | 129,265 |
| 13 | (TS=(“Physical Activity”)) OR TS=(“Physically Active”) | 232,706 |
| 14 | (((((((TS=(“Screen Time”)) OR TS=(“Screen Behavior”)) OR TS=(“Screen Behaviour”)) OR TS=(“Screen Use”)) OR TS=(“Screening Time”)) OR TS=(“Screening Behavior”)) OR TS=(“Screening Behaviour” )) OR TS=(“Screening Use”) | 7,360 |
| 15 | (((((TS=(“Computer Time”)) OR TS=(“Internet Time”)) OR TS=(“Computer Use”)) OR TS=(“Internet Use”)) OR TS=(“Computer Usage”)) OR TS=(“Internet Usage”) | 18,189 |
| 16 | OR/10-15 | 465,824 |
| 17 | TS=(Random*) | 2,226,082 |
| 18 | ((((TS=(Blind*)) OR TS=(Singleblind*)) OR TS=(Doubleblind*)) OR TS=(Trebleblind*)) OR TS=(Tripleblind*) | 545,376 |
| 19 | (TS=(RCT*)) OR TS=(Control*) | 7,278,894 |
| 20 | ((((TS=(“Clinical Trial”)) OR TS=(“Clinical Trials”)) OR TS=(“Clinical Study”)) OR TS=(“Clinical Studies”)) OR TS=(“Intention to Treat Analysis”) | 636,810 |
| 21 | OR/17-20 | 9,207,101 |
| 22 | #9 AND #16 AND #21 | 8,910 |

Database: EMBASE <1 March 2023>

Search Strategy:

| **#** | **Searches** | **Results** |
| --- | --- | --- |
| 1 | 'occupation'/exp OR 'occupation' | 447,736 |
| 2 | 'workplace'/exp OR 'workplace' | 82,251 |
| 3 | 'employee'/exp OR 'employee' | 49,198 |
| 4 | 'office'/exp OR 'office' | 351,061 |
| 5 | 'worker'/exp OR 'worker' | 147,910 |
| 6 | 'staff'/exp OR staff | 389,707 |
| 7 | 'white collar worker'/exp OR 'white collar worker' | 727 |
| 8 | occupation:ti,ab,kw OR occupational:ti,ab,kw | 233,037 |
| 9 | workplace:ti,ab,kw OR workplaces:ti,ab,kw | 66,264 |
| 10 | employee:ti,ab,kw OR employees:ti,ab,kw | 70,157 |
| 11 | office:ti,ab,kw OR offices:ti,ab,kw | 118,206 |
| 12 | 'work site':ti,ab,kw OR 'work sites':ti,ab,kw OR worksite:ti,ab,kw OR worksites:ti,ab,kw | 5,909 |
| 13 | worker:ti,ab,kw OR workers:ti,ab,kw | 271,793 |
| 14 | staff:ti,ab,kw | 271,7144 |
| 15 | 'white collar worker':ti,ab,kw | 76 |
| 16 | OR/1-15 | 1,594,090 |
| 17 | 'sedentary lifestyle'/exp OR 'sedentary lifestyle' | 23,308 |
| 18 | 'sitting'/exp OR 'sitting' | 49,090 |
| 19 | 'inactivity'/exp OR 'inactivity' | 27,663 |
| 20 | 'physical activity'/exp OR 'physical activity' | 592,002 |
| 21 | 'screen time'/exp OR 'screen time' | 5,458 |
| 22 | sedentary:ti,ab,kw | 51,215 |
| 23 | sitting:ti,ab,kw | 37,854 |
| 24 | inactivity:ti,ab,kw OR inactive:ti,ab,kw | 147,831 |
| 25 | 'physical activity':ti,ab,kw OR 'physically active':ti,ab,kw | 199,221 |
| 26 | 'screen time':ti,ab,kw OR 'screen behavior':ti,ab,kw OR 'screen behaviour':ti,ab,kw OR 'screen use':ti,ab,kw OR 'screening time':ti,ab,kw OR 'screening behavior':ti,ab,kw OR 'screening behaviour':ti,ab,kw OR 'screening use':ti,ab,kw | 7,569 |
| 27 | 'computer time':ti,ab,kw OR 'internet time':ti,ab,kw OR 'computer use':ti,ab,kw OR 'internet use':ti,ab,kw OR 'computer usage':ti,ab,kw OR 'internet usage':ti,ab,kw | 8,379 |
| 28 | OR/17-27 | 797,458 |
| 29 | 'randomized controlled trial'/exp OR 'randomized controlled trial' | 1016,756 |
| 30 | 'randomized controlled trial':ti,ab,kw | 139,853 |
| 31 | randomized:ti,ab,kw | 931,120 |
| 32 | blind:ti,ab,kw OR blinds:ti,ab,kw OR blinded:ti,ab,kw OR blinding:ti,ab,kw OR singleblind:ti,ab,kw OR doubleblind:ti,ab,kw OR trebleblind:ti,ab,kw OR tripleblind:ti,ab,kw | 445,053 |
| 33 | rct:ti,ab,kw OR rcts:ti,ab,kw OR controlled:ti,ab,kw | 1,247,596 |
| 34 | 'clinical trial':ti,ab,kw OR 'clinical trials':ti,ab,kw OR 'clinical study':ti,ab,kw OR 'clinical studies':ti,ab,kw OR 'intention to treat analysis':ti,ab,kw | 916,916 |
| 35 | OR/29-34 | 2,829,984 |
| 36 | #16 AND #28 AND #35 | 6,928 |
