# Supplementary material for: Effectiveness of Multicomponent Interventions in Office-Based Workers to Mitigate Occupational Sedentary Behavior: Systematic Review and Meta-Analysis
Source: JMIR Public Health Surveill. 2023 Jul 26;9:e44745. doi: 10.2196/44745 (PMC10413238; doi:10.2196/44745)
Supplement: Multimedia Appendix 2 [file publichealth_v9i1e44745_app2.docx]

**Multimedia Appendix 2.** Basic characteristics of the included studies

| References | First author (year), Country | Study design | Sample (n) | Age | Follow-up point^d^ | Installment of the sit-stand workstation | Dropouts^e^ | Measurement tool | Outcomes (unit) |
| --- | --- | --- | --- | --- | --- | --- | --- | --- | --- |
| [15] | Danquah et al (2017), Denmark | Cluster RCT^a^ | I^b^: 141 | 46 (10) | 1-month; 3-month | Yes | 6.80% | ActiGraph GT3X+ | Sitting/Standing/Prolonged sitting time (min/8-h workday) |
|  |  |  | C^c^: 115 | 45 (11) |  |  |  |  |  |
| [35] | Edwardson et al (2018), England | Cluster RCT | I: 77 | 41.7 (11.0) | 3-month; 6-month; 12-month | Yes | 24.66% | activPAL monitor | Sitting/Standing/Stepping/Prolonged sitting time (min/workday) |
|  |  |  | C: 69 | 40.8 (11.3) |  |  |  |  |  |
| [34] | Edwardson et al (2022), England | Cluster RCT | I: 489 | 44.9 (10.0) | 3-month; 12-month | Yes | 31.42% | activPAL3 monitor | Sitting/Standing/Stepping/Prolonged sitting time (min/work hours) |
|  |  |  | C: 267 | 44.5 (11.2) |  |  |  |  |  |
| [36] | Engelen et al (2019), Australia | Quasi-RCT | I: 23 | 44.48 | 6-week; 13-week | Yes | 21.74% | ActiGraph GT3X+ | Sitting/Standing/Stepping time (proportion of workday) |
|  |  |  | C: 13 |  |  |  |  |  |  |
| [13] | Healy et al (2013), Australia | Quasi-RCT | I: 18 | 42.4 (10.6) | 4-week | Yes | 16.28% | activPAL3 monitor | Sitting/Standing/Stepping/Prolonged sitting time (min/8-h workday) |
|  |  |  | C: 18 | 42.9 (10.3) |  |  |  |  |  |
| [37] | Healy et al (2016), Australia | Cluster RCT | I: 96 | 44.6 (9.1) | 3-month; 12-month | Yes | 13.42% | activPAL3 monitor | Sitting/Standing/Stepping/Prolonged sitting time (min/8-h workday) |
|  |  |  | C: 65 | 47.0 (9.7) |  |  |  |  |  |
| [38] | Lin et al (2018), Taiwan | Quasi-RCT | I: 50 | 49.5 | 3-month; 12-month | No | 1.98% | self-efficacy scale | Sitting time (h/day) |
|  |  |  | C: 48 |  |  |  |  |  |  |
| [39] | Maylor et al (2018), England | Cluster RCT | I: 38 | 43.0 (12.8) | 8-week | No | 21.84% | activPAL monitor | Sitting/Standing/Stepping/Prolonged sitting time (min/workday) |
|  |  |  | C: 30 | 43.7 (13.1) |  |  |  |  |  |
| [14] | Neuhaus et al (2014), Australia | Quasi-RCT | I: 12 | 37.3 (10.7) | 3-month | Yes | 16.67% | activPAL3 monitor | Sitting time (min/8-h workday) |
|  |  |  | C: 13 | 48 (11.6) |  |  |  |  |  |
| [40] | Nooijen et al (2020), Sweden | Cluster RCT | I: 106 | 41 (9) | 6-month | Yes | 35.52% | Actigraph GT3X | Sitting/Standing/Stepping time (percnet of average work time) |
|  |  |  | C: 61 | 44 (8) |  |  |  |  |  |
| [41] | Renaud et al (2020), the Netherlands | Cluster RCT | I: 92 | 43.0 (10.3) | 4-month; 8-month | No | 20.90% | activPAL monitor | Sitting/Standing/Stepping/Prolonged sitting time (h/8-h workday) |
|  |  |  | C: 92 | 41.5 (10.1) |  |  |  |  |  |

^a^RCT: randomized controlled trial

^b^I: intervention

^c^C: control

^d^Follow-up point: results at the primary endpoint are used in the meta-analysis when there are multiple assessment points

^e^Dropouts (%): dropout rate at the primary endpoint
