# Supplementary material for: Effectiveness of Multicomponent Interventions in Office-Based Workers to Mitigate Occupational Sedentary Behavior: Systematic Review and Meta-Analysis
Source: JMIR Public Health Surveill. 2023 Jul 26;9:e44745. doi: 10.2196/44745 (PMC10413238; doi:10.2196/44745)
Supplement: Multimedia Appendix 3 [file publichealth_v9i1e44745_app3.docx]

**Multimedia Appendix 3.** Detailed account of the constituent elements and implementation particulars of the multicomponent interventions implemented in each of the included trials.

| References | | Study | Strategies | Interventions | Detailed information |
| --- | --- | --- | --- | --- | --- |
| [15] | Danquah et al, 2017 | | Organizational strategies | **Appointment of ambassadors** | Each office appointed an ambassador to provide social support, and to make sure the project and the common goals were discussed regularly. |
|  |  | |  | **Management support** | Further, managers agreed to act as role models. To ensure commitment, meetings were held with management and ambassadors before the workshop and after 1 month. |
|  |  | |  | **Lecture** | Aimed at increasing participants’ knowledge of sedentary behavior and health, a 15-min lecture communicated recent evidence and allowed time for questions. The lecture was given at the start of the workshop (described below). Lecture content was also communicated in a leaflet and via the project website. |
|  |  | |  | **Workshop** | Aimed at ensuring local adaptation at individual, office and workplace level. The workshop was conducted once at each workplace and was held during work hours by two of the researchers. Participants were guided through four strategies: using a sit-stand desk, breaking up prolonged periods of sitting, having standing and walking meetings and setting common goals at office level. For each strategy, participants were given examples of changes they could implement, and examples were discussed. Individual goals included for example, raising the table each morning, or moving the waste paper basket away from the table. Common goals included implementing a standing office meeting and reminding one another to raise the table to the standing position. Individual goals were noted on a personal card. Common goals were written on posters which were put up in the office together with general posters displaying the examples given. Additionally, participants received a sticky-notes block and a postcard to remind them of the project. The content of the workshop was the same at each workplace, but goals were different, depending on personal and local circumstances. However, many of the selected common goals were similar, e.g. most offices decided to have standing-meetings but exactly which meetings or whether only part of the meeting should take place standing depended on the local meeting structure, duration and facilities. |
|  |  | | Individual strategies | **E-mails** | Participants could volunteer to receive weekly e-mails and/or twice-weekly text messages during the intervention period. The content varied according to the four strategies from the workshop and reiterated some of the examples. |
|  |  | |  | **Text messages** | Participants could volunteer to receive weekly e-mails and/or twice-weekly text messages during the intervention period. The content varied according to the four strategies from the workshop and reiterated some of the examples. |
|  |  | | Environmental strategies | **Work environmental changes** | Each intervention office installed high meeting tables in meeting rooms, offices and corridors. Additionally, routes for walking meetings were defined. |
|  |  | |  | **Individual sit-stand workstation** | Sit-stand desks were in place |
| [35] | Edwardson et al, 2018 | | Organizational strategies | **Management support** | We sought management buy-in by meeting with the chief executive of the hospital trust. He showed his support for the study and the intervention through his regular e-newsletter sent to all staff, and through members of the Clinical Management Groups who were also asked to show support (ie, encourage involvement and allow time for intervention activities) and to filter this message down to the other management team leads. |
|  |  | |  | **Education seminar** | An initial group-based education seminar (around 30 minutes’ duration) was delivered, which covered the health consequences of sitting and the benefits of reducing and regularly breaking up sitting. These messages were also reinforced in a leaflet provided at the end of the seminar. Participants were given their baseline results from the activPAL device at the end of the seminar, which informed them of their sitting (total and prolonged), standing, and stepping time at work, and overall daily levels. |
|  |  | |  | **Coaching sessions** | To provide ongoing support to participants, a trained member of the research team offered brief (about 15 minutes) coaching sessions, either face-to-face or by telephone, at month 1 and every three months thereafter to discuss progress, review goals and action plans, and discuss personal or social and group barriers and any benefits experienced. |
|  |  | | Individual strategies | **Action plan** | They were then provided with an action plan and goal setting booklet and encouraged to set a goal around sitting less at work based on their activPAL feedback and to create an action plan for this to be achieved. |
|  |  | |  | **Promotion** | We provided participants with a DARMA cushion (Darma, CA, USA). to enable them to more regularly track and self-monitor their sitting time (total and prolonged) and be prompted (in the form of a vibration) to regularly break up sitting. This cushion, which can be placed on an office chair, is approximately 2.5 cm thick and uses Bluetooth to sync data with a mobile phone app to provide the participant with real-time feedback. The frequency of the vibration prompt is a user defined setting (e.g., can be set up to vibrate every 30 or 45 minutes). Every few months the participants received posters, with either educational or motivational messages. |
|  |  | |  | **Feedback** | After each visit for follow-up measurements, the participants were provided with their results from the activPAL device, and these were compared with the baseline data. This allowed the participants to review their progress and goals. |
|  |  | | Environmental strategies | **Individual sit-stand workstation** | Participants were provided with a height adjustable desk or desk platform to enable them to sit or stand to work. They were given a choice between a full sized electric desk (twin leg single step stand desk 1200×800, MACOI, Kimbolton, UK), which allows the desk top to move up and down, or a choice of two sizes of desk platform (Pro Plus 30 or Pro Plus 48, VARIDESK; TX), which sits on the existing desk allowing the computer screen and keyboard to be moved up and down. This choice allowed flexibility for office set-up and to avoid testing the effectiveness of a specific type of desk rather than the height adjustable desk concept. We provided a brief training session on how to use the desk or platform and on the ergonomic set-up. A leaflet was also provided to reinforce these messages. |
| [34] | Edwardson et al, 2022 | | Organizational strategies | **Management support** | Support of senior leaders was secured through a series of business case documents and videos, which articulated the importance of reducing employee sitting behaviors, the positive impact this reduction could have on workplace culture, and how reduction in sitting time might be achieved without disrupting performance and productivity. Workplace champions, who were council employees enrolled as participants in the study, were identified within each cluster and facilitated the interventions. Workplace champions attended a training session (three hours) delivered by a behavior change education team to equip them with the skills and knowledge for facilitation of the interventions. The senior management team within each council allowed workplace champions protected time each month for facilitation of the interventions. |
|  |  | |  | **Education session** | Workplace champions provided participants with a link to a one-off online education session that covered the adverse health consequences of excessive sitting and reinforced the benefits of breaking up sitting time and reducing overall sitting time. The session also encouraged participants to estimate their own sitting time at work and at home and to think about strategies to reduce and break up sitting time in both environments; provided a range of ideas on how to reduce and break up sitting time at work and at home; covered identification of barriers, and goal setting; provided information on the importance of self-monitoring and prompts for behavior change; and suggested a range of free smartphone enabled applications and computer software or extensions to use at work and home. |
|  |  | |  | **Group catch-up sessions** | Group catch-up sessions were encouraged at three and nine months to provide an opportunity for participants to collectively review key messages of the intervention, brainstorm ideas, discuss any barriers and facilitators to reducing sitting time, and develop new goals and action plans. |
|  |  | | Individual strategies | **Action plan** | Participants were provided with a range of downloadable resources, including posters, ideas to reduce and break up sitting, and an action plan and goal setting sheet. The main message of the intervention was to sit less (<50% of the waking day spent sitting) and move more often (every 30 minutes). |
|  |  | |  | **Challenges** | Sitting less and moving more challenges were provided that could be completed individually, in groups, or with family and friends, with the suggestion to take part in these challenges three times during the 12-month study period. |
|  |  | | Environmental strategies | **Work environmental changes** | The intervention promoted small scale restructuring of the office environment (e.g., relocation of printers and wastepaper bins, standing meetings, standing areas in shared space) to encourage more frequent movement around the office. Participants were also encouraged to think about their home environment. Motivational posters were embedded in the office environment. The workplace champions demonstrated positive examples within the working environment (e.g., behavioral modelling). |
|  |  | |  | **Individual sit-stand workstation** | Clusters randomized to the SWAL plus desk group received a height adjustable desk to encourage them to transition between sitting and standing postures while working. Participants were able to select their preferred desk type and colour (black or white) from four models: Deskrite 100 (Posturite, Berwick, UK), Yo-Yo Desk Mini, Yo-Yo Desk 90, or Yo-Yo Desk Go (Sit-Stand Trading, Swindon, UK). All the desks were designed to sit on top of the participants’ existing workstation. Participants were provided with an information booklet and guidance from the research team on how to use the desk appropriately when in the sitting and standing positions, as well as recommendations based on the sedentary office expert statement on how much standing to achieve throughout the day (e.g., gradually work towards two hours of standing and light movement and eventually towards four hours). The importance of avoiding prolonged standing was also highlighted. |
| [36] | Engelen et al, 2019 | | Organizational strategies | **Workshop** | In week 1 of the intervention, participants in the intervention group were invited to attend a one-hour workshop hosted by the organization. Researchers presented the risks of prolonged sitting and benefits of breaking up sitting time, followed by researcher-facilitated group discussions and brainstorming on how sitting less and moving more could be promoted and realistically implemented in their workplace. |
|  |  | |  | **Site Visits** | Three site visits were conducted by the research team, which coincided with each assessment period. These visits included visual assessment of sit-stand desk setups and advice given to participants where required. Site visits provided employees with an opportunity to discuss barriers, questions and difficulties relating to standing and to the broader research project. In some cases this included modifying the approach to standing i.e. switching from using the timer to using a task based approach such as standing while checking emails, in other instances participants had questions about wearing their activity monitors or wanted to share positive anecdotal feedback. During these site visits the researcher also met with the organization’s Workplace Health and Safety officer, who relayed anecdotal feedback and other issues raised by the participants. |
|  |  | | Individual strategies | **Prompting app** | Participants downloaded a free application to their desktop computer that prompts users to stand or sit, based on the user-determined preferences. The app’s default timer setting was 20 minutes continuous standing and 40 minutes sitting. Researchers instructed participants to adjust the timer setting to meet their personal circumstances and gradually increase the time standing as suitable. Participants were advised that once they felt comfortable with the standing time to increase their standing time by 2-5 minutes per session. Participants were also advised of the potential adverse health impacts of prolonged standing and researchers recommended not exceeding 60 minutes continuous standing time. At the end of the day the app gives the users the opportunity for self-monitoring, by providing a brief report on the overall time they spent sitting and standing. This visual and auditory reminder raises awareness and the daily report reinforces healthy behavior change.1The app was an optional intervention component. It was downloaded by 75% of the participants, but only used by 32%, where instead some participants preferred to intersperse short standing breaks (for 2-3 minutes), every 20 minutes; while others preferred to take a task-based approach and for example stand while they were talking on the phone. |
|  |  | |  | **Weekly emails** | Intervention participants received a welcome email with tips and guidelines to increasing standing at work and a short informative YouTube video on the health risks of prolonged sitting. Subsequently, participants received weekly emails with helpful Move More, Sit Less strategies, videos and reminders to use their height adjustable desk. Researchers used content and materials produced by the Heart Foundation NSW, The Blue Earth Foundation and ASAP Science. |
|  |  | | Environmental strategies | **Individual sit-stand workstation** | The intervention involved the employer providing participants with a sit-stand desks (Varidesk Models, Pro 30 and Pro Plus 36). Upon receiving the sit-stand desk, participants received brief training from the organization’s Workplace Health and Safety officers on how to use the sit-stand desk correctly and safely, including how to adjust workstation height, safe ergonomic set ups while sitting and standing, and how to gradually increase standing time to prevent injury and fatigue. |
| [13] | Healy et al, 2013 | | Organizational strategies | **Management support** | The intervention began with a 45-minute researcher-led consultation with unit representatives from the intervention group and management. This provided background information and emphasized the importance of organizational support for successful intervention adoption. Representatives brainstormed and selected organization-specific strategies to the “Stand Up, Sit Less, Move More” approach. A liaison person from the organization (LG) served as the interface between the research team and the organization, with regular contact serving to troubleshoot any difficulties and reinforce active organizational support. Additionally, the liaison person sent two emails (template provided by research team) noting study progress and providing a “standing tip of the week”. |
|  |  | |  | **Workshop** | The research team then conducted a workshop for all intervention participants, providing further intervention details and information on the health consequences of excessive sitting, and discussing as well as adjusting the identified strategies. |
|  |  | | Individual strategies | **Coaching sessions** | Two master's level health coaches delivered an initial 30-minute face-to-face consultation with each intervention participant, followed by three telephone calls (one/week). Wherever possible, employees spoke to the same coach. These sessions emphasized behavior change strategies (goal-setting, self-monitoring, and use of prompts and problem solving: (Abraham and Michie, 2008)), consistent with the motivational interviewing approach utilized. |
|  |  | |  | **Feedback** | The coaching session included feedback on participants’ baseline activity and monitor results, which were used to inform personally tailored goals regarding each of the intervention messages. Specific recommendations encouraged participants to: Stand Up at least every 30 min; Sit Less by using the workstation, aiming for approximately equal amounts of sitting and standing time, but emphasizing the need for gradual increases and frequent postural change; and, Move More by increasing incidental physical activity. |
|  |  | | Environmental strategies | **Individual sit-stand workstation** | Dual display sit-stand workstations, including the work surface accessory, were provided and installed for the 4-week duration of the study (installation duration ~45 min/workstation). The workstations allowed employees to alternate their posture between sitting and standing. Participants received brief verbal instructions from research staff and an information sheet, which included ergonomic guidelines from the manufacturer. An occupational therapist (provided by Comcare) was available for further advice and follow-up assessments. |
| [37] | Healy et al, 2016 | | Organizational strategies | **Management support** | An initial consultation with senior management established the departmental resources available to support the program at an organizational level. Then, a three-hour group consultation workshop with representatives from each of the intervention sites (managers, team champions, occupational health and safety representatives and general staff) was held to inform management and other organizational stakeholders about the study’s broad aims and discuss the feasibility of the study from a management and team perspective. At this workshop, a range of organizational-level strategies appropriate for the various intervention sites were brainstormed. These strategies, as well as the baseline feedback, were then subsequently discussed with all participants at each intervention worksite to identify those strategies most suitable to their work context, with the site-specific strategies finalized using a participatory approach. Team champions (typically the worksite team leader) were recruited and encouraged to role-model and promote the organizational-level strategies. This included sending six emails, which the champion could tailor to include messages relevant for their team, at weeks 2, 4, 6, 8, 10, and 12. Research staff were copied in on the emails for monitoring of intervention fidelity. |
|  |  | | Individual strategies | **Coaching session** | These consisted of an individual face-to-face coaching session (0-3 days following workstation installation) at the participants’ workplace and four telephone calls at weeks 2, 4, 8 and 12. The coaching was used to: explain the “Stand Up, Sit Less, Move More” intervention targets; indicate the extent to which participants were meeting these targets according to their baseline assessment results; and, to identify specific goals and individual-level behavior change strategies relating to each of these key intervention messages. Participants recorded their goals and strategies on their personal tracker, which was affixed to their workstation. During the face-to-face coaching session, participants also received specific instructions to listen to their body, and to regularly change posture (i.e., to neither sit nor stand for too long). |
|  |  | |  | **Promotion** | Following the consultation, a personalized email summary of the session was sent to participants. The telephone calls were used to support goal attainment. The calls involved assessment of participant progress toward previously set goals, problem-solving as necessary, and adjustment/progression of goals and related behavior change strategies. The telephone call at week eight focused on sitting and activity outside of the workplace. Intervention fidelity was maintained through the health coach’s use of detailed intervention scripts and checklists and quarterly meetings with senior study investigators. |
|  |  | | Environmental strategies | **Individual sit-stand workstation** | A dual-screen sit-stand workstation, with a work surface accessory, was installed for the duration of the study (12 months). Participants received written and verbal instructions and tips on the appropriate ergonomic posture for both sitting and standing, as recommended by the product manufacturer, as well as adhesive stickers applied by research staff to indicate the recommended configuration tailored for each individual (i.e., appropriate desk height when standing/sitting). |
| [38] | Lin et al, 2018 | | Organizational strategies | **Pedometer challenge** | 1. Each intervention participant received a pedometer and Participant Handbook. 2. Participant Handbook described the intervention content and timing, benefits and incentives, and participant responsibilities (including measures). 3. Description of the Pedometer Challenge: (1) benefits, (2) how to use the pedometer, (3) how to set personal goal and team goal, (4) support teams, (5) incentives, and (6) how to record steps and sitting time daily using Step Log and Sitting Time Log. 4. Individual goals were targeted toward > 10,000 steps a day. 5. Team goal achievement: every member of a team that had 75% of its members meet or exceed target goals. The number one team received prizes from the company’s senior management. 6. Team approach: participants were divided into 8 teams (4–9 members/team), and each team selected a team leader. Team leaders were responsible for encouraging participants to achieve goals and win prizes for their team, as well as serving as coordinators between participants and research liaison. Team leaders helped collect Step Log and Sitting Time Log biweekly from every team member. 7. A large poster, presented in a break room, was designed to record and compare team goal achievement and was updated biweekly. |
|  |  | |  | **Walking route and resources** | 1. A walking route was designed in the workplace. 2. Signage boards that displayed around the walking route were used to convey information about direction, essential walking posture tips, walking details (including distance, pace, speed, duration, and calories burned), and inspirational messages to keep their motivation going. 3. Walking maps close to the company and participants’ home were provided |
|  |  | | Individual strategies | **Monthly newsletters** | Three monthly newsletters were created: 1. Exercise more to promote health: introducing benefits of being active and risks of physical inactivity, and ways to increase physical activity in the workplace and daily life 2. Sitting too much is bad for your health, let’s get moving: describing health risks of prolonged sitting and moving more to promote health 3. How much physical activity do you need: recommending type, intensity, frequency, duration, and total amount of physical activity |
|  |  | |  | **Motivational tools** | Six motivational tools adapted were: 1. Setting goals 2. Overcoming barriers 3. Sedentary temptations 4. Avoiding relapse 5. Staying motivated 6. Keep on moving |
|  |  | | Environmental strategies | **Work environmental changes** | 1. Prompting posters with either positive (e.g., encouragements) or negative (e.g., warnings) messages were displayed throughout the workplace where employees made choices or where they definitely saw them. 2. Prompting posters also loaded onto participants’ office computers to remind them to take a break for 1 minute every 1 hour. 3. Prompting posters were interesting and changed biweekly to attract participants’ attention. |
| [14] | Neuhaus et al, 2014 | | Organizational strategies | **Management support** | The organizational intervention addressed some aspects of workplace culture and norms via inclusion of a consultation with the unit manager, an all-of-staff information session, and manager e-mails to employees. The manager consultation (~30 minutes) provided the rationale for the study, details of participation, and a discussion of approaches to stand up, sit less, and move more within their unit. Over the course of the intervention, six fortnightly e-mails were sent from the manager to staff. They supported program participation, and included a study information booklet (provided by research staff). The remaining five e-mails encouraged staff to stand up, sit less, and move more and commented on strategies that appeared to be working well within the unit. E-mail templates were provided by research staff and tailored to the group by the manager. |
|  |  | |  | **Information and brainstorming session** | The ensuing 30-minute staff information and brainstorming session addressed the study rationale and procedures, as well as feedback on the unit’s baseline workplace sitting time. |
|  |  | | Individual strategies | **Face-to-face coaching** | The initial 30-minute face-to-face coaching session was delivered at the worksite within 2 days following the workstation installation. This included a discussion of graphic feedback on the individual’s baseline sitting, standing, and moving time and collaborative goal-setting in relation to the three program messages. |
|  |  | |  | **E-mails** | An e-mail summarizing the agreed-upon goals was sent to each participant on the same day. |
|  |  | |  | **Telephone support** | Three follow-up telephone calls (~10 minutes each) were delivered at 1, 3, and 7 weeks following the coaching session to assess goal achievement, problem-solve potential barriers, and reset goals as necessary. |
|  |  | |  | **Feedback** | Participants also received a laminated self-monitoring tool. This Tracker was attached to the workstation, clearly visible to the participant and used during the coaching session and telephone calls for the participant to document and adjust specific goals and strategies used. Participants were able to contact the project manager at any time in the case of adverse events or problems with their workstation. |
|  |  | | Environmental strategies | **Individual sit-stand workstation** | The environmental intervention strategy modified the personal physical office environment through the provision of fully installed height-adjustable workstations (WorkFit-S) with an attached work surface tray for each intervention participant. Employees also received verbal (10-minute duration) and written instructions from the project manager on correct usage and how to alternate their working posture in line with OHS guidelines. |
| [39] | Maylor et al, 2018 | | Organizational strategies | **Educational presentation** | Following baseline assessments, all intervention participants received an educational presentation from the project team informed by scientific evidence on the dangers of excessive sitting and the benefits of interrupting sitting time. A summary of these strategies was subsequently emailed to all intervention participants by the project team the following work day. |
|  |  | |  | **Brainstorming session** | Participants then took part in a brainstorming session to identify and agree upon strategies to reduce sitting within their workplace. A summary of these strategies was subsequently emailed to all intervention participants by the project team the following work day. |
|  |  | |  | **Step challenge** | Immediately following the educational presentation and brainstorming session, each participant was provided with a pedometer, goal setting guidance (provided during individual meetings described below), and took part in a step challenge during the intervention period. Each participant entered their daily steps onto a virtual leaderboard and spot prizes (shopping gift vouchers) were provided to increase motivation. |
|  |  | | Individual strategies | **Health check report** | One week after the educational presentation, participants were provided with a personal health check report during a ~20 min face-to-face meeting with a member of the project team. The report was generated from Health Options v9.1.31 software (Health Diagnostics Ltd, Chester, UK), which is designed for use within National Health Service Health Check programmers. The report provides risk scores and educational information on diabetes, cholesterol, cardiovascular disease, and weight management. |
|  |  | |  | **Individual meetings** | During the individual meeting, each participant received a goodie bag that contained a leaflet briefly outlining the intervention procedures, a facts sheet on the dangers of prolonged sitting, an information card on “what your steps mean” (i.e. the number of daily steps equating to low active, moderately active, active, and highly active), sticky notes to place around their workspace with self-selected reminders to encourage less sitting, and a prompt card to remind participants of sitting reduction strategies. |
|  |  | |  | **Promotion** | Participants received instructions to download computer software (Break Timer, Tom Watson, Spain) and/or a phone app from a list provided (e.g. Rise & Recharge, Baker Heart and Diabetes Institute, Australia; Break Reminder, TheBigMom, USA) that prompted them to get up and move at regular intervals. Participants were advised to set the regularity of the alerts according to their own personal preference. Point of decision poster prompts were also displayed around the working environment (e.g., office walls, notice boards, and near lifts) encouraging employees to interrupt their sitting time and increase their steps. |
|  |  | |  | **Telephone support** | One-to-one telephone support (5-10 min) was provided weekly from a member of the project team during intervention weeks 2 to 8 and followed a semi-structured script to maintain intervention fidelity. The telephone calls were based on motivational interviewing and involved discussions around participant progress toward goals, problem-solving, and adjustment of goals and behavior change strategies as necessary. |
|  |  | | Environmental strategies | **Work environment changes** | Participants were asked to make changes to their working environment in line with strategies identified during the brainstorming session. Examples of these strategies included removal or relocation of personal bins and printers, and identification of workspaces or meeting areas to be used specifically for non-computer based work to encourage movement away from the desk. |
| [40] | Nooijen et al, 2020 | | Organizational strategies | **Management support** | (1) Intervention to promote physical activity level: team leaders encouraged employees to be physically active inside and outside of working hours, including commuting to work. (2) Targeting sedentary behavior: team leaders encouraged employees to reduce sedentary behavior during work, in meetings, and while sitting behind their desks as well as outside of working hours including lunch and commuting. |
|  |  | | Individual strategies | **Motivational counselling** | (1) Intervention to promote physical activity level: using cognitive behavioral therapy towards increasing time spent in moderate-to-vigorous intensity physical activity, including individual feedback on physical activity. (2) Targeting sedentary behavior: using cognitive behavioral therapy towards reducing time in sedentary behavior and breaking up prolonged sitting, including individual feedback on sedentary behavior patterns. |
|  |  | | Environmental strategies | **Work environmental changes** | Intervention to promote physical activity level: access to a commercial gym (6 months), exercise sessions and lunch walks organized by team leaders, and the provision of company bikes |
|  |  | |  | **Individual sit-stand workstation** | Targeting sedentary behavior: team leaders were instructed to organize standing and walking meetings. Note that companies already provided their employees with sit-stand desks. |
| [41] | Renaud et al, 2020 | | Organisational components | **Management support** | Managers of each participating department had an initial face-to-face meeting with the internal occupational physiotherapist. During this meeting, the potential motivating role of the manager (e.g., leading by example) in the context of the Dynamic Work intervention was discussed. Two team meetings were planned with the occupational physiotherapist. These meetings lasted 30 min and were scheduled approximately four weeks apart. In the first team meeting, the health risks associated with prolonged sitting and correct (ergonomic) usage of the new furniture and other components were explained. During the second team meeting, experiences with the sit-stand workstations, the desk bikes, the sit balls and the individual activity/sitting tracker (see below) were discussed with a focus on overcoming any barriers to decreased sitting time, increased standing time or steps that were experienced. |
|  |  | | Individual components | **Consultations** | During the intervention period, the occupational physiotherapists proactively approached participants to inquire about their experience during on-site consultations (at least two per department). These consultations took place on the open-plan office floors, were generally of short duration, had an ad hoc nature, and were intended to provide tips and reflect on individual questions of participants about the Dynamic Work intervention (e.g., questions regarding ergonomic position, personal goals, and dealing with barriers). |
|  |  | |  | **Activity/sitting tracker** | In collaboration with the researchers the Activator, a novel, individual activity/sitting tracker (PAL technologies, Glasgow, UK) was provided to all participants in the intervention group. This tracker provided users with feedback on step count, time spent upright and percentage of time spent sitting via a smartphone app. The device had an option to set haptic vibration feedback after 15 or 30 min of uninterrupted sitting. Additionally, participants received a 12-week, self-help “sit less, move more” programmer booklet, in which personal goals could be set. Use of the Activator and the booklet were explained during the first group meeting with the occupational physiotherapist. |
|  |  | | Environmental component | **Shared sit-stand workstation** | The initial plan was to place electrically adjustable sit-stand workstations (VEPA, Hoogeveen, The Netherlands) in the open-plan, shared office spaces of participating departments in a one-to-three ratio with regular, sitting workstations. Because of the extra departments included, the actual ratio changed to one-to-four, on average, as a fixed number of workstations had to be shared between more employees (due to financial constraints) |
|  |  | |  | **Shared desk bikes and sit balls** | Furthermore, desk bikes (Work trainer, Harmelen, The Netherlands) were provided for every second sit-stand workstation, and at least two office sit balls were provided per intervention department (Vluvstuf, Breda, The Netherlands). |
