# Supplementary material for: Effectiveness of Multicomponent Interventions in Office-Based Workers to Mitigate Occupational Sedentary Behavior: Systematic Review and Meta-Analysis
Source: JMIR Public Health Surveill. 2023 Jul 26;9:e44745. doi: 10.2196/44745 (PMC10413238; doi:10.2196/44745)
Supplement: Multimedia Appendix 4 [file publichealth_v9i1e44745_app4.docx]

**Multimedia Appendix 4.** Subgroup analyses of multicomponent intervention effects on the mitigation of occupational sedentary behavior based on the installation of sit–stand workstations, feedbacks, and prompts.

| Outcomes | Subgroup | | | | | |
| --- | --- | --- | --- | --- | --- | --- |
|  | Whether to install the sit-stand workstation | | Whether feedback | | Whether prompt | |
|  | Installment of the sit-stand workstation | No installment of the sit-stand workstation | Feedback | No feedback | Prompt | No prompt |
| Occupational sitting time^a^ | -71.95 (-92.94, -51.15) | -14.25 (-22.78, -5.72) | -67.48 (-122.55, -12.41) | -46.19 (-69.56, -22.81) | -45.31 (-68.57, -22.04) | -59.17 (-96.29, -22.05) |
| *P* value | .001 | .001 | .02 | .001 | .001 | .001 |
| I^2^ | 84.2% | 0.0% | 91.8% | 89.4% | 80.5% | 93.6% |
| Occupational standing time^a^ | 66.56 (43.45, 89.67) | 6.82 (-0.09, 13.67) | 60.15 (-10.34, 130.63) | 39.31 (16.55, 62.07) | 33.86 (12.34, 55.38) | 54.65 (17.62, 91.68) |
| *P* value | .001 | .06 | .10 | .001 | .01 | .01 |
| I^2^ | 89.9% | 0.0% | 95.2% | 92.3% | 83.3% | 95.5% |
| Occupational stepping time^a^ | 1.53 (-0.90, 3.96) | 5.22 (0.31, 10.13) | -0.78 (-4.43, 2.88) | 5.31 (0.97, 9.66) | 5.03 (-2.77, 12.84) | 2.30 (0.05, 4.55) |
| *P* value | .22 | .04 | .68 | .02 | .21 | .045 |
| I^2^ | 5.8% | 74.9% | 0.0% | 72.5% | 81.3% | 0.8% |
| Prolonged occupational sitting time^a^ | -47.05 (-73.66, -20.43) | -11.49 (-22.78, -0.19) | -33.00 (-71.24, 5.25) | -33.31 (-58.48, -8.13) | -12.74 (-22.38, -3.10) | -60.76 (-89.49, -32.03) |
| *P* value | .001 | .04 | .10 | .01 | .01 | .001 |
| I^2^ | 88.7% | 0.0% | 78.7% | 88.7% | 0.0% | 79.3% |

^a^min/8-h workday, mean difference (95% confidence interval)
