# Supplementary material for: Effectiveness of Multicomponent Interventions in Office-Based Workers to Mitigate Occupational Sedentary Behavior: Systematic Review and Meta-Analysis
Source: JMIR Public Health Surveill. 2023 Jul 26;9:e44745. doi: 10.2196/44745 (PMC10413238; doi:10.2196/44745)
Supplement: Multimedia Appendix 5 [file publichealth_v9i1e44745_app5.docx]

| **Multimedia Appendix 5.** Summary of findings for multicomponent interventions for mitigating occupational sedentary behavior among office-based workers. | | | | | | |
| --- | --- | --- | --- | --- | --- | --- |
| **The multicomponent intervention compared to no intervention for occupational sedentary behavior** | | | | | | |
| **Patient or population: office-based workers**  **Setting: workplace**  **Intervention: the multicomponent intervention**  **Comparison: no intervention** | | | | | | |
| Outcomes | Anticipated absolute effects^*^ (95% CI) | | Relative effect (95% CI) | № of participants (studies) | Certainty of the evidence (GRADE) | Comments |
|  | Risk with placebo | Risk with Change in sedentary time |  |  |  |  |
| Occupational sitting time |  | MD **52.25 lower** (73.06 lower to 31.44 lower) | - | 1894 (11 RCTs) | ⨁⨁⨁◯ Moderate^a^ |  |
| Occupational standing time |  | MD **44.30 higher** (23.11 higher to 65.48 higher) | - | 1869 (10 RCTs) | ⨁⨁⨁◯ Moderate^a^ |  |
| Occupational stepping time |  | MD **3.14 higher** (0.19 lower to 6.47 higher) | - | 1515 (8 RCTs) | ⨁⨁⨁◯ Moderate^a^ |  |
| Occupational prolonged sitting time |  | MD **32.63 lower** (51.93 lower to 13.33 lower) | - | 1568 (7 RCTs) | ⨁⨁⨁◯ Moderate^a^ |  |
| ***The risk in the intervention group** (and its 95% confidence interval) is based on the assumed risk in the comparison group and the **relative effect** of the intervention (and its 95% CI). **CI:** confidence interval; **MD:** mean difference | | | | | | |
| **GRADE Working Group grades of evidence** **High certainty:** we are very confident that the true effect lies close to that of the estimate of the effect. **Moderate certainty:** we are moderately confident in the effect estimate: the true effect is likely to be close to the estimate of the effect, but there is a possibility that it is substantially different. **Low certainty:** our confidence in the effect estimate is limited: the true effect may be substantially different from the estimate of the effect. **Very low certainty:** we have very little confidence in the effect estimate: the true effect is likely to be substantially different from the estimate of effect. | | | | | | |

#### Explanations

a. Downgraded one level because studies were considered as high risk of bias due to the allocation sequence was not randomized, there was possible contamination between the intervention and control groups, self-reported outcome measures, and there was a high rate of loss to follow-up and unclear risk of bias due to no information about random allocation sequence generation and trial registration or published protocol.
